# Traumatic Events and PTSD Among Palestinian Children and Adolescents: The Effect of Demographic and Socioeconomic Factors

**DOI:** 10.3389/fpsyt.2020.00004

**Published:** 2020-03-31

**Authors:** Basel El-Khodary, Muthanna Samara, Chris Askew

**Affiliations:** ^1^Department of Psychology, Kingston University London, Kingston upon Thames, United Kingdom; ^2^Department of Psychology, University of Surrey, Guildford, United Kingdom

**Keywords:** war traumatic events, Posttraumatic Stress Disorder, Palestinian, children, adolescents, socioeconomic status

## Abstract

**Background:**

The situation in the Gaza Strip is uncommon in the frequency with which children are exposed to war-related traumatic events on a daily basis and because of the long-term nature of the conflict. The prevalence of posttraumatic stress disorder (PTSD) among children and adolescents in the Gaza Strip increased after the recent wars. The aims of the study are: To investigate the prevalence and nature of war traumatic events and PTSD; and to investigate how these traumatic events predict PTSD when taking into account demographic and socioeconomic status factors amongst Palestinian children and adolescents in the Gaza Strip.

**Methods:**

The sample consists of 1,029 school pupils (11–17 years old): 533 (51.8%) were female and 496 (48.2%) were male. War-Traumatic Events Checklist (W-TECh) Post-Traumatic Stress Disorders Symptoms Scale (PTSDSS) were used.

**Results:**

The majority of children and adolescents experienced personal trauma (*N*: 909; 88.4%), witnessed trauma to others (*N*: 861; 83.7%) and observed demolition of property (*N*: 908; 88.3%) during the war. Compared to girls, boys showed significantly more exposure to all three event types as well as overall traumatic events. Results also demonstrated that the prevalence of DSM-V PTSD diagnosis was 53.5% (*N* = 549). Further, children who had experienced personal trauma, trauma to others, and the demolition of property were significantly more likely to be diagnosed with PTSD compared to those who had not, even when adjusting for demographic and socioeconomic factors. The strongest war trauma for PTSD is personal trauma followed by witnessing trauma and then observing demolition of properties.

**Conclusions:**

The study provides valuable evidence that demographic and socioeconomic factors mediate the relationship between different war traumatic events and PTSD. Interventions should take into account the children’s background including their gender, age, where they live, and their socioeconomic status (e.g., family income, parents' educational level, family size) to alleviate the psychological symptoms and to enhance their resilience.

## Introduction

A large number of children live in conditions of political violence, terrorist, and war situations worldwide (1). War-related stressors may include shelling, bombing, home demolition, and exposure to the wounding and killing of family members or loved ones (2). As a result, children may have feelings of unsafety and altered daily functioning when they are exposed to war-traumatic events (3). Moreover, children and adolescents growing up in situations of political violence and terrorism are vulnerable to damaging developmental consequences (4) and intense psychological effects (5–8); these, in turn, can lead to psychiatric symptomatology (7, 9–11).

Children do not develop in isolation, they both actively shape and are shaped by the social worlds in which they live. The ecological theory highlights the development of the child within his/her environment and the interaction emerged between the two of them. This interaction consists of a social network or a variety of contexts or ecologies that shape the child's personality and behaviors (12, 13) and play a major role in their development (12, 14–18). Beside the individual characteristics of the child (e.g., gender, age, family order), family (e.g., family size, socioeconomic status), and environment (e.g., war traumatic events) appear to be a primary context in order to understand the development of PTSD.

Several studies have revealed that exposure to previous traumatic war experiences and events is a risk factor for the development of post-traumatic stress disorder (PTSD), grief, and depression (21, 22). The exposure to traumatic events, specifically physical injuries, loss of loved ones, immediate risk of life (2, 23), injury of a family member or friend (2, 24–26) and losing a family member (27) are the strongest risk factors for PTSD.

Demographic characteristics such as age, gender, type of residence, and socioeconomic status have also been found to be related to PTSD. Findings for age have been varied, while several studies showed that older children exhibit more symptom of PTSD than younger children (e.g., 28), other studies have shown the opposite (e.g., 29) or even no difference (e.g., 30). A similar discrepancy is seen in results describing gender differences: Some studies report that females show more PTSD symptoms (e.g., 31) while others have either found that males exhibit more symptoms (5) or found no differences (e.g., 30). Children who live in cities report less PTSD than those in villages (30) and those with low socioeconomic status report more psychological distress including anxiety, depression, and PTSD (e.g., 5, 26). For adolescents, having an unemployed father is a risk factor for PTSD, anxiety, and depression compared to having a father in employment (28).

The situation in the Gaza Strip is uncommon in the frequency with which children are exposed to war-related traumatic events on a daily basis and because of the long-term nature of the conflict. Altawil et al., (32) found that every single child had been exposed to three or more traumatic events. In addition, 42% were suffering from moderate or acute PTSD levels. Another study showed that 54.7% of Palestinian children have been exposed to at least one traumatic event in their life. Of these, 49% have experienced a war-related trauma (5). Furthermore, the results of an assessment of the impact of war-trauma on adolescents in the Gaza Strip and South Lebanon by Khamis (26) indicated that around 30% of adolescents have been exposed to war traumatic events during their lives. Adolescents in the Gaza Strip showed more PTSD compared to the adolescents in south Lebanon.

Exposure to political violence and war in Palestine began with the 1948 war between the Jewish and the Arab armies. As a result of this war, many Palestinians were killed and their villages destroyed with half of Palestinians becoming refugees either in the West Bank or Gaza, or in Arab countries, such as Lebanon, Jordan and Syria. In 1967, the Israeli army further occupied the West Bank and the Gaza Strip. In December 1987, the first Palestinian uprising (Intifada) began and Palestinians in the occupied territories (the Gaza Strip and the West Bank) commenced a revolution against the Israeli occupation. The second uprising started in 2000 and more sophisticated weapons, such as tanks and airplanes, were used by the Israeli army. The number of people killed and wounded increased accordingly. Hence, the prevalence of PTSD and other mental health problems also increased (33). In December 2008, the Israeli army launched a war against the Gaza Strip, which was more severe than the first and second uprisings. Palestinians were attacked using rockets from the air, tanks on the ground, and military ships. This war continued for 22 days, and more than 1,400 Palestinians were killed with mass destruction of houses and farms (34).

Throughout the Israeli attacks against civilians on the Gaza Strip that took place from 14–26 November 2012, 175 civilians were killed. Of them, 59 were children and 11 were women. A further 1,399 civilian people were wounded, of them 606 were children and 254 were women (35). There are few studies on the effects of long-term exposure to war-traumatic events on the children and adolescents in the Gaza Strip (27). The current study provides a unique opportunity to investigate a large sample of children who have experienced a chronic war trauma over time in a conflict situation. The study aims to explore the prevalence of PTSD symptoms and diagnosis according to DSM-V among children and adolescents after the war conducted against civilians in the Gaza Strip in November 2012. Also, we aim to examine the different risk factors and consequences of PTSD in children and adolescents in the Gaza Strip and identify the high-risk groups for PTSD. We hypothesize that risk factors such as exposure to war-traumatic events, and demographic variables such as age, gender, type of residence, socioeconomic status may be related to PTSD among Palestinian children in the Gaza Strip.

## Methods

### Participants and Procedures

Palestinian school children and adolescents (7^th^, 8^th^, and 10^th^ grade; *N* = 1,131) aged 11–17 years old (*M* = 13.71, *SD* = 1.36) were approached to participate in the study. All parents agreed that their children take part in the study. Of these, 102 students were absent at the time of data collection or transferred to another school. As a result, the total number of the sample was 1,029 students; 533 (51.8%) were female and 496 (48.2%) were male. The participants were chosen according to place of residence (Rafah, Khan Younis, Middle Area, Gaza, or North Gaza), type of school (primary or secondary), and gender (male or female) using a stratified random sampling. From each place of residence, two types of schools (one primary and one secondary) were randomly chosen; then from these schools, one male school and one female school were randomly chosen, and then, one class from each single school was selected randomly. Finally, 30 classes, 10 classes from each grade (7^th^, 8^th^, and 10^th^ grades) were chosen on the basis of five male classes and five female classes. Thirty social workers and school counselors were fully trained by the researchers and performed the interviews with the children and adolescents in the chosen schools in the Gaza Strip. The data were collected in October 2013 one year after the Israeli attacks on Gaza which occurred between the periods 14^th^ to 26^th^ November 2012.

### Ethical Procedure

Children were given an information sheet about the study and a parental consent form to give to their parents. Ethical approval was gained from the Ministry of Education in the Gaza Strip and from the ethical committee of the Faculty of Business and Social Sciences (FBSS) at Kingston University London.

### Study Instruments

Data collection in the form of interviews with children and adolescents was conducted in schools. Two separate sessions were held; each of them lasted approximately 40 minutes.

*Demographic variables* included age (11–17 years), which was categorized to three groups: (1) youngest age group: 11–12 years, (2) middle age group: 13–14 years, and (3) oldest age group: 15 years or older; gender (male, female); family order (the first, the middle, the last sibling); family size (below or above six members) as the average house hold size in the Gaza Strip is six members (36); type of residence (city, camp, village); parents' education (have not attended any school education, school education, higher education (e.g., university)); parents' job (employed, unemployed); citizenship (refugee, not refugee); whether parents are alive or dead; and monthly family income (below or above US $600) (36).

*War-Traumatic Events Checklist (W-TECh):* The W-TECh was constructed by two of the authors (19; 20, 37). Some of the items were adapted from Gaza Traumatic Events Checklist (38) and the Trauma Questionnaire Scale (39). The measure was modified to include items that reflect the last traumatic war events that happened in the Gaza Strip. It consists of 28 items that cover war-traumatic experiences such as human traumatic losses or injuries and home demolition. To ensure that the answers are specific to the 2012 war, the data collectors asked the participants to answer the items based on their experiences during the 2012 war.

The W-TECh includes three categories: (1) experiencing personal trauma, in which children or adolescents were the target of war-related traumas such as being shot or injured with live ammunition; (2) witnessing human trauma, in which children or adolescents witnessed others (e.g., family member, friend, or neighbor) being shot and/or injured during the war; and (3) seeing demolition of property, in which children or adolescents observe the demolition of their home, school, and/or farm during the war. Children and adolescents responded to the W-TECh by indicating whether they experienced the traumatic event or not.

*Post-Traumatic Stress Disorders Symptoms Scale (PTSDSS) (*32): The scale consists of 50 items and has been modified to include the diagnostic criteria of PTSD according to DSM-5 (40), which includes intrusion symptoms, avoidance, negative alterations in cognitions and mood, and alterations in arousal and reactivity. Functional impairment included items related to somatic symptoms (e.g., “I get tired easily”) cognitive symptoms (e.g., “I cannot stop thinking about the traumatic event that I was exposed to”), emotional symptoms (e.g., “I get tense and nervous easily without good reason”), social symptoms (e.g., “I like to break the rules of my family or school”), and academic dysfunction symptoms (e.g., “I cannot concentrate on my study”). Children and adolescents rate their experiences on a 5-point Likert scale (*very often*, *often*, *moderately*, *few*, or *never*).

Participants were considered to have PTSD when they had a) been exposed to at least one war traumatic event as measured by the W-TECh; b) scored moderately to very often for at least one intrusion symptom, at least one avoidance symptom, at least two negative alterations in cognitions and mood symptoms, and at least two symptoms related to alterations in arousal and reactivity; c) significant alteration in functional impairment; and d) symptoms duration of more than 1 month (40).

### Statistical Analysis

*T-tests* were used to investigate the difference between two variables (e.g., male and female), one-way ANOVA to examine the difference among three or more variables (e.g., type of residence: city, camp, or village), and chi-square analysis to investigate the difference between two or more categorized variables (e.g., type of residence and PTSDD). Pearson correlation coefficients analysis was performed to examine the association of war-traumatic events categories (exposure to personal trauma, witnessing trauma to others, and seeing demolition of properties) and PTSD criteria, and functional impairments. Furthermore, linear and logistic regression analyses were employed in order to examine the association between PTSD as the dependent (outcome) variable and demographic variables (e.g., age, gender) and traumatic events categories (exposure to personal trauma, witnessing trauma to others, and seeing demolition of properties) as the independent (predictor) variables.

## Results

### Demographics Factors

The age of the participants was 11–17 years. Of them, 51.8% were females, and the family size ranged from 2 to 18 (*M* = 8.6, *SD* = 2.41). The majority of the participants are middle children in their families (64.5%), live in the city (67.2%), and the majority (94.7%) had their parents alive (see **Table 1**).

**Table 1:** Demographic variables frequencies by total trauma and PTSD.

	*N*	%	Total trauma *M* (*SD*)	PTSD % (*N*)
**Age** 12 or less 13-14 15 or more	184 485 344	17.9 47.1 33.4	*p* <.001 7.93 (4.85) 8.74 (4.93) 10.33 (4.42)	*p* = .03 46.2 (85) 57.4 (278) 53.4 (183)
**Gender** Male Female	496 533	48.2 51.8	*p* <.001 10.12 (4.66) 8.20 (4.87)	*p = *.525 52.4 (259) 54.4 (290)
**Family size** Below average Above average	149 793	14.5 77	*p = *.192 8.59 (4.40) 9.14 (4.82)	*p = *.171 48.3 (72) 54.4 (431)
**Family order** The first The middle The last	199 664 160	19.3 64.5 15.5	*p = *.144 9.27 (4.62) 8.94 (4.79) 9.75 (5.18)	*p = *.306 52.8 (105) 54.8 (363) 48.1 (77)
**Place of residence** North Gaza Gaza Middle area Khan Younis Rafah area	217 201 184 198 228	21.1 19.5 17.9 19.2 22.1	*p* <.001 10.84 (4.51) 10.88 (3.87) 8.55 (4.78) 8.50 (5.51) 6.98 (4.10)	*p = *.209 57.6 (125) 57 (114) 53.8 (99) 47.2 (93) 51.8 (118)
**Type of residence** City Refugee camp Village	692 132 203	67.2 12.8 19.7	*p = *.003 8.88 (4.80) 8.82 (5.16) 10.18 (4.53)	*p = *.221 54.1 (374) 46.6 (61) 55.7 (113)
**Family income** Below average Above average	770 217	74.8 21.1	*p = *.03 9.26 (4.82) 8.48 (4.89)	*p = *.245 54.7 (420) 50.2 (109)
**Father education** None School education Higher education	190 543 280	18.4 52.7 27.2	*p = *.02 9.43 (4.82) 9.30 (4.82) 8.35 (4.64)	*p = *.01 55.8 (106) 55.7 (302) 44.2 (96)
**Father job** Unemployed Employed	469 548	45.5 53.2	*p = *.252 9.31 (4.98) 8.96 (4.67)	*p = *.464 54.5 (255) 52.2 (286)
**Father** Alive Dead	989 37	96.0 3.6	*p = *.04 9.07 (4.77) 10.70 (5.86)	*p = *.05 52.8 (522) 69.4 (25)
**Mother education** None School education Higher education	176 624 219	17.1 60.6 21.3	*p = *.07 9.59 (5.36) 9.19 (4.67) 8.51 (4.74)	*p = *.189 52.3 (92) 55.5 (346) 48.2 (92)
**Mother job** Unemployed Employed	948 73	92.0 7.1	*p = *.423 9.16 (4.75) 8.63 (5.54)	*p = *.02 54.5 (516) 41.1 (30)
**Mother** Alive Dead	998 25	96.9 2.4	*p = *.56 9.12 (4.84) 9.54 (3.45)	*p = *.79 53.4 (532) 56 (14)
**Citizenship** Refugee Not refugee	335 690	32.5 67.0	*p = *.003 9.76 (4.47) 8.82 (4.94)	*p = *.89 53.1 (178) 53.6 (369)

### Prevalence of War Traumatic Events and PTSD

The results demonstrated that every child or adolescent has experienced at least one war-traumatic event (*M* = 9, *SD* = 4.8). The most prevalent war-traumatic events were witnessing or hearing shelling by tanks, artillery, or military planes (90.4%, *N* = 931); witnessing the signs of shelling on the ground (75%, *N* = 766); and witnessing the injury or killing of someone by the occupied forces (58.7%, *N* = 605). The least prevalent war traumatic events were shooting with live ammunition by the occupied forces (3%, *N* = 31), being injured (e.g., wounds, burns, or bone break) by shelling, tanks, artillery, or military planes (4%, *N* = 41); and being beaten by the occupied forces (5%, (*N* = 49). When looking at the categories of the traumatic events, the results revealed that about 88.3% (*N* = 909) experienced personal trauma, 83.7% (*N* = 861) witnessed trauma to others, and 88.2% (*N* = 908) had seen demolition of properties during the war (see **Table 2**).

**Table 2:** War traumatic events frequency.

No.	Items	N	%
1	Has your house been destroyed completely by shelling or bulldozing?	59	5.7
2	Has your house been destroyed partially by shelling or bulldozing?	17	16.9
3	Have you been shot with live ammunition by occupied forces?	31	3
4	Have you been injured (e.g., wounds, burns, or bone break) by shelling, tanks, artillery, or military planes?	41	4
5	Have you been injured to the degree that you lost consciousness?	68	6.6
6	Have you been exposed to live fire by occupied forces, but you were not injured?	101	9.8
7	Have you been beaten by occupied forces?	49	4.8
8	Have you been exposed to shelling by tanks, artillery, or military planes, but you were not injured?	456	44.3
9	Have the occupied forces sieged your house, block, camp, or zone?	154	15
10	Has someone of your family members been killed by occupied forces?	85	8.3
11	Has someone of your friends, neighbours, or close relatives been killed by occupied forces?	389	37.8
12	Has someone of your family members been injured by occupied forces?	107	10.4
13	Has someone of your friends, neighbours, or close relatives been injured by occupied forces?	337	32.7
14	Have you attended to martyr's funeral?	380	36.9
15	Have you been forced to leave your home during the war?	328	31.8
16	Have you been detained at home during incursion?	367	35.6
17	Have you been threatened to death by being used as human shield to arrest your neighbours by the army?	73	7.1
18	Have you witnessed the occupied forces destroying house(s)?	552	53.6
19	Have you witnessed or hearing shelling by tanks, artillery, or military planes?	931	90.4
20	Have you witnessed or hearing the occupied forces opening fire against people?	442	42.9
21	Have you witnessed the occupied forces beating some one?	472	45.8
22	Have you witnessed killing or injuring someone by the occupied forces?	605	58.7
23	Have you witnessed arresting someone by the occupied forces?	426	14.4
24	Have you witnessed the occupied forces destroying trees and farms?	491	47.7
25	Have you witnessed the occupied forces were not allowed Ambulance access to hospital?	396	38.4
26	Have you witnessed the signs of shelling on the ground?	766	74.4
27	Have you watched mutilated bodies?	579	56.2
28	Have you witnessed assassination of people by rockets?	489	47.2

Of the entire sample, 54% (*N* = 549) met the PTSD criteria of DSM-V; 87% (*N* = 896) had intrusion symptoms, 59.8% (*N* = 616) had avoidance symptoms, 86.2% (*N* = 888) had negative alterations in cognitions and mood, and 76.9% (*N* = 792) had alterations in arousal and reactivity. Moreover, about 46% (*N* = 468) suffered from moderate to severe somatic symptoms; 68.4% (*N* = 704) suffered from moderate to severe cognitive symptoms; 68.6% (*N* = 707) suffered from moderate to severe emotional symptoms; 52.2% (*N* = 538) suffered from moderate to severe social symptoms, and 52.4% (*N* = 539) suffered from symptoms of moderate to severe academic dysfunction.

### Demographic Variables and Exposure to War-Traumatic Events

The relationship of various factors with exposure to traumatic war events was investigated. One-way ANOVA indicated that the effect of age was significant, *F*(2,1006) = 18.45, *p* < .001, $\eta_{p}^{2}$ = .035. LSD *post hoc* tests revealed that the oldest age group showed more exposure to overall war-traumatic events than the youngest age group (*p* < .001) and middle age group (*p* < .001). There was also a significant effect of gender among children and adolescents, *t*(1,020.27) = 6.50, *p* < .001, *d = *.402 with boys showing more exposure to traumatic war events overall compared to girls (see **Table 1**).

Type of residence was found to have a significant effect on war-traumatic events experienced, *F*(2,1020) = 6.01, *p* < .001, $\eta_{p}^{2}$ = .012. LSD *post hoc* tests showed that children and adolescents who lived in villages experienced more of these events than those who lived in cities (*p = *.001) and those who live in refugee camps (*p = *.01). There were also significant differences related to family income, *t*(981) = 2.10, *p = *.03, *d = *.160. Children and adolescents from families who earned less than the average income (US $550) reported higher exposure to war-related traumatic events overall than those whose families earned more than the average income (see **Table 1**).

Fathers' education level was also found to have a significant effect on exposure to war trauma, *F*(2,943) = 3.55, *p = *.02, $\eta_{p}^{2}$ = .007. LSD *post hoc* tests showed that children and adolescents whose fathers attended higher education (e.g., university) were exposed to less overall war-traumatic events than those whose fathers had no school education (*p = *.02), and those whose fathers attended school education (*p = *.01). There was also a significant difference between those whose fathers were alive and those whose fathers were dead, *t*(1020) = 2.02, *p = *.04, *d* = −.305. Children and adolescents whose fathers were dead reported more exposure to overall war-traumatic events compared to those whose father's were still alive (*p = *.04). Finally, the results indicated that there was a significant effect of citizenship, *t*(1019) = 3.02, *p* < .001, *d = *.180. Refugee children and adolescents reported more exposure to war traumatic events overall compared to non-refugee ones (see **Table 1**).

### Demographic Variables and PTSD Diagnosis

The results showed that levels of PTSD diagnosis according to DSM-V significantly differed across age groups, χ²(2, *N* = 1,011) = 6.87, *p = .03*, with middle age group children meeting PTSD criteria more often than the youngest and oldest age groups (see **Table 1**). Results also showed a significant association between children's and adolescents' fathers' education and PTSD, χ²(2, *N* = 949) = 8.88, *p = *.01. Children and adolescents whose fathers attended school education were more likely to develop PTSD than those whose fathers attended higher education (e.g., university), and those whose fathers have no schooling. In addition, a significant association was found between PTSD diagnosis and the mothers of children and adolescents' employment status, χ²(1, *N* = 1,020) = 4.88, *p = *.02: unemployed mothers of children and adolescents showed a diagnosis of PTSD more often than employed mothers. There was also a significant difference in frequency of PTSD diagnosis between those with a living or dead father, χ²(1, *N* = 1,024) = 3.85, *p = *.05: children and adolescents whose fathers were dead had a PTSD diagnosis more often than those whose fathers were still alive (see **Table 1**).

Pearson's correlation analyses were performed to investigate associations between several continuous demographic variables (age, family size, family income, and parent's education) and both PTSD criteria (intrusion symptoms, avoidance, negative alterations in cognitions and mood, and alteration in arousal and reactivity) and functional impairment (somatic symptoms, cognitive, emotional, social, and academic dysfunction symptoms). Results indicated that age was positively correlated with increased arousal and reactivity, social symptoms, and symptoms of academic dysfunction (see **Table 3**): older children show higher levels of arousal and reactivity, social symptoms, and symptoms of academic dysfunction. There was also a positive relationship between family size and both PTSD criteria and functional impairment symptoms; so that the greater the size of the family, the greater was the level of PTSD criteria and functional impairment symptoms. In contrast, family income was found to be negatively correlated with PTSD criteria (except for avoidance, which was nonsignificant) and functional impairment symptoms. Children with high family income showed lower symptoms of PTSD criteria (except avoidance) and functional impairment symptoms. Similarly, parent's education level was negatively associated with intrusion symptoms, alterations in arousal and reactivity, somatic symptoms, social symptoms, and academic dysfunction symptoms, so that children with more highly educated parents had less of these symptoms.

**Table 3:** Correlations between exposure to traumatic events categories, PTSD total score, PTSD criteria, and functional impairments symptoms.

	1	2	3	4	5	6	7	8	9	10	11	12	13	14	15	16	17	18	19
1. Age	-																		
2. Family size	.023	-																	
3. Family income	.015	-.123^**^	-																
4. Father education	.000	−.101^**^	.382^**^	-															
5. Mother education	−.066^*^	−.114^**^	.304^**^	.524^**^	-														
6. Personal Trauma	.129^**^	.057	−.020	−.103^**^	−.096^**^	-													
7. Witnessing trauma to others	.200^**^	.072^*^	−.044	−.049	−.050	.501^**^	-												
8. Seeing properties demolition	.168^**^	.084^**^	−.005	−.041	−.033	.552^**^	.720^**^	-											
9. Overall trauma	.203^**^	.081^*^	−.031	−.075^*^	−.073^*^	.772^**^	.906^**^	.847^**^	-										
10. PTSD	.059	.124^**^	−.102^**^	−.063^*^	−.075^*^	.361^**^	.273^**^	.280^**^	.354^**^	-									
11. Intrusion symptoms	−.012	.128^**^	−.115^**^	−.093^**^	−.075^*^	.195^**^	.159^**^	.189^**^	.207^**^	.741^**^	-								
12. Avoidance symptoms	.049	.069^*^	−.003	−.020	−.034	.246^**^	.186^**^	.193^**^	.241^**^	.654^**^	.490^**^	-							
13. Negative alterations in cognitions and mood	.041	.074^*^	−.070^*^	−.037	−.056	.350^**^	.270^**^	.253^**^	.342^**^	.880^**^	.609^**^	.584^**^	-						
14. Alterations in arousal and reactivity	.097^**^	.115^**^	−.092^**^	−.062^*^	−.088^**^	.331^**^	.246^**^	.237^**^	.318^**^	.842^**^	.560^**^	.565^**^	.759^**^	-					
15. Somatic symptoms	−.001	.122^**^	−.133^**^	−.091^**^	−.094^**^	.298^**^	.251^**^	.224^**^	.307^**^	.801^**^	.673^**^	.531^**^	.706^**^	.700^**^	-				
16. Cognitive symptoms	.046	.104^**^	−.081^*^	−.032	−.031	.303^**^	.260^**^	.267^**^	.324^**^	.856^**^	.664^**^	.508^**^	.775^**^	.685^**^	.702^**^	-			
17. Emotional symptoms	−.006	.112^**^	−.074^*^	−.034	−.011	.164^**^	.184^**^	.214^**^	.224^**^	.785^**^	.761^**^	.459^**^	.640^**^	.586^**^	.667^**^	.688^**^	-		
18. Social symptoms	.076^*^	.087^**^	−.081^*^	−.067^*^	−.084^**^	.389^**^	.238^**^	.209^**^	.326^**^	.838^**^	.484^**^	.663^**^	.752^**^	.798^**^	.663^**^	.638^**^	.485^**^	-	
19. Academic dysfunction symptoms	.115^**^	.083^*^	−.099^**^	−.101^**^	−.135^**^	.358^**^	.242^**^	.234^**^	.324^**^	.750^**^	.383^**^	.428^**^	.733^**^	.757^**^	.575^**^	.568^**^	.384^**^	.702^**^	-

**Correlation is significant at the.01 level (2-tailed).

*Correlation is significant at the.05 level (2-tailed).

Finally, significant positive correlations were found between exposure to war-traumatic events categories, PTSD criteria (intrusion symptoms, avoidance, negative alterations in cognitions and mood, and alterations in arousal and reactivity), and functional impairment symptoms (somatic, cognitive, emotional, social, and academic dysfunction symptoms). The more children and adolescents exposed to war-traumatic events, the more they met PTSD criteria and showed symptoms of functional impairment (see **Table 3**).

### Prediction of PTSD

To investigate predictors of PTSD total score symptoms (adding the items of PTSD together), each of the demographic variables was first entered alone into a series of simple univariate linear regression models (see **Table 4**). Variables showing significant prediction of PTSD were gender (female), *F*(1, 1,025) = 6.77, *p = *.009, large family size; *F*(1, 939) = 14.610, *p = *.000; low family income, *F*(1, 983) = 10.34, *p = *.001; low father education, *F*(1, 1,010) = 4.03, *p = *.04; low mother education *F*(1, 1,016) = 5.67, *p = *.01; village residence, *F*(1, 1,023) = 6.465, *p = *.01; exposure to personal trauma, *F*(1, 1,024) = 153.91, *p* <.001; witnessing trauma to others, *F*(1, 1,024) = 82.32, *p* < .001, seeing properties demolished, *F*(1, 1,024) = 87.40, *p* <.001; and overall war-related traumatic events, *F*(1, 1,021) = 146.32, *p* <.001.

**Table 4:** Linear regression: prediction of PTSD from demographic variables and traumatic events.

Predictors	Univariate	Step 1	Step 2	Step 3	Step 4	Step 5
	β [95% CI]	β [95% CI]	β [95% CI]	β [95% CI]	β [95% CI]	β [95% CI]
**Demographic variables**						
Age	.05 [−.03, 2.09]	.09 [.27, 2.98]	.06 [−.21, 2.30]	.05 [−.38, 2.26]	.06 [−.29, 2.33]	.04 [−.58, 1.98]
Gender (Female)	−.08 [.94, 6.75] *	.06 [−.34, 6.14] *	.17 [5.24, 11.53] ***	.09 [1.33, 7.66] **	.08 [.72, 7.01] *	.13 [3.24, 9.49] ***
Family income	−.10 [−3.41, −.82] *	−.07 [−3.21,.03]	−.07 [−3.00,.00] *	−.06 [−2.89,.25]	−.07 [−3.17, −.03] *	−.06 [−2.88,.17]
Family size	.12 [.58, 1.81] ***	.11 [.46, 1.78] **	.07 [.08, 1.31] *	.09 [.25, 1.53] **	.09 [.232, 1.51] **	.07 [.11, 1.37] *
Father education	−.06 [−1.92, −.02] *	−.02 [−1.62,.88]	−.001 [−1.18, 1.14]	−.01 [−1.44,.98]	−.01 [−1.41, 1.00]	−.007 [−1.28, 1.07]
Mother education	−.07 [−2.29, −.22] *	−.04 [−2.14,.54]	−.04 [−1.92,.57]	−.04 [−2.08,.51]	−.05 [−2.17,.43]	−.04 [−1.98,.55]
Father’s job	−.03 [−4.34, 1.52]	.07 [−.16, 7.12]	.07 [.01, 6.78] *	.06 [−.45, 6.60]	.06 [−.42, 6.62]	.06 [−.53, 6.33]
Mother's job	−.05[−10.34,.94]	.008 [−5.56, 7.05]	−.006 [−6.36, 5.35]	.01 [−4.68, 7.51]	.01 [−4.92, 7.26]	.01 [−4.92, 6.92]
Father's alive	.036 [−3.21, 12.59]	.03 [−4.35, 15.55]	.005 [−8.61, 9.93]	.03 [−4.19, 15.06]	.03 [−5.26, 13.97]	.02 [−5.57, 13.19]
Mother's alive	−.008 [−10.60, 8.28]	−.01 [−13.39, 7.86]	.01 [−7.91, 11.87]	−.01 [−12.82, 7.73]	−.009 [−11.59, 8.95]	−.008 [−11.58, 8.91]
Type of residence (city)	−.03 [−4.62, 1.58]					
Type of residence (refugee camp)	−.05 [−8.07,.631]	−.05 [−8.73, 1.14]	−.05 [−8.64,.51]	−.02 [−6.24, 3.37]	−.04 [−7.56, 1.99]	−.02 [−6.10, 3.23]
Type of residence (Village)	.07 [1.07, 8.36] *	−.01 [−4.83, 3.65]	−.02 [−5.50, 2.38]	−.02 [−5.55, 2.67]	−.02 [−5.52, 2.69]	−.02 [−5.56, 2.42]
Citizenship	−.008 [−3.49, 2.71]	.004 [−3.59, 3.97]	.004 [−3.29, 3.72]	.02 [−2.41, 4.92]	.01 [−3.02, 4.28]	.02 [−2.30, 4.84]
**Exposure to war-traumatic events**						
Personal trauma	.36 [3.87, 5.33] ***		.39 [4.23, 5.91] ***			
Witnessing trauma of others	.27 [1.95, 3.03] ***			.26 [1.78, 2.97] ***		
Seeing properties demolition	.28 [3.59, 5.50] ***				.26 [3.17, 5.24] ***	
Overall trauma	.35 [1.46, 2.03] ***					.35 [1.44, 2.08] ***
***R*^2^**		0.04	0.174	0.103	0.105	0.154

***p < .001, **p < .01, *p < .05.

Next, a regression model was created with demographic variables entered together in step 1. This indicated that gender (being female) and having larger family size significantly predicted PTSD even when adjusting for the other factors entered in the model. Whether demographic variables moderate the effect of exposure to personal trauma on PTSD was investigated in step 2. Gender (female), low family income, large family size, and unemployed fathers significantly moderated the effect of exposure to personal trauma. In step 3, demographic variables' moderation of the effect of witnessing trauma to others on PTSD was examined. Being female and from large family size were significant moderators of the effect of witnessing trauma to others. Moderation effects of demographic variables' on the effect of seeing properties demolished on PTSD was investigated in step 4. Being female, from low family income, and large family size significantly moderated the effect of seeing properties demolished. Finally, in step 5, the regression model investigated whether demographic variables moderate the effect of exposure to overall trauma on PTSD. Being female and from large family size were found to be significant moderators of this effect. The highest *R^2^* that explains PTSD is the model that included personal trauma (17.4%) followed by total trauma (15.4%), witnessing trauma of others (10.3%) and demolition of properties (10.5%) (see **Table 4**).

Logistic regression analysis was performed to examine the association between exposure to war-traumatic events and PTSD diagnosis according to DSM-V moderated by demographic variables. When demographic variables were entered alone, analyses indicated that low father education (*p = *.01), unemployed mother (*p = *.02), exposure to personal trauma (*p* <.001), witnessing trauma to others (*p* <.001), seeing properties demolished (*p* < .001), and exposure to overall trauma (*p* < .001) significantly predicted PTSD diagnosis (Univariate analysis) (see **Table 5**). Next, demographic variables were entered together. In step 1, being an older child and having a father with low education significantly predicted PTSD. In step 2, moderation by demographic variables of the effect of exposure to personal trauma on PTSD diagnosis was investigated. Being an older child, being females, and living in a city significantly moderated the effect of children and adolescents exposure to personal trauma. Whether demographic variables moderated the effect of witnessing trauma to others on PTSD diagnosis were analyzed in step 3. Being older and having a father with low education level significantly moderated the effect of children and adolescents witnessing trauma to others. Step 4 investigated whether demographic variables moderate the effect of seeing properties demolished on PTSD diagnosis. Being an older child and lower father's education significantly moderated the effect of seeing properties demolished. Finally, demographic variables' moderation of the effect of exposure to overall trauma on PTSD diagnosis was examined in step 5. Being an older child, female, and living in a city significantly moderated effects of exposure to overall trauma. The highest *R^2^* that explains PTSD is the model that included personal trauma (12.3%) followed by total trauma (11.1%), witnessing trauma of others (8.1%) and demolition of properties (9%) (see **Table 5**).

**Table 5:** Logistic regression: prediction of PTSD from demographic variables and traumatic events.

Predictors	Univariate	Step 1	Step 2	Step 3	Step 4	Step 5
	Exp(B) [95% CI]	Exp(B) [95% CI]	Exp(B) [95% CI]	Exp(B) [95% CI]	Exp(B) [95% CI]	Exp(B) [95% CI]
**Demographic variables**						
Age	1.08 [.99, 1.18]	1.21 [1.07, 1.37]**	1.18 [1.03, 1.34]*	1.17 [1.03, 1.33]*	1.17 [1.03,1.33]*	1.15 [1.01, 1.31]*
Gender (Female)	1.08 [.84, 1.38]	1.16 [.86, 1.56]	1.59 [1.15, 2.19]**	1.26 [.93, 1.70]	1.21 [.90, 1.64]	1.40 [1.02, 1.91]*
Family income	.83 [.61, 1.13]	.88 [.59, 1.31]	.94 [.62, 1.41]	.95 [.63, 1.42]	.92 [.61, 1.38]	.96 [.64, 1.44]
Family size	1.27 [.89, 1.81]	1.16 [.79, 1.72]	1.08 [.72, 1.61]	1.12 [.76, 1.67]	1.13 [.76, 1.67]	1.10 [.74, 1.64]
Father education	.78 [.64,.95]*	.76 [.58,.98]*	.78 [.59, 1.01]	.76 [.58,.98]*	.76 [.58,.99]*	.77 [.59, 1.00]
Mother education	.91 [.74, 1.12]	.93 [.70, 1.23]	.95 [.71, 1.27]	.94 [.71, 1.25]	.95 [.71, 1.26]	.95 [.71, 1.27]
Father’s job	.91 [.71, 1.16]	1.22 [.89, 1.66]	1.24 [.90, 1.71]	1.21 [.88, 1.66]	1.20 [.87, 1.65]	1.21 [.88, 1.66]
Mother’s job	.58 [.35,.94]*	.58 [.29, 1.13]	.54 [.27, 1.09]	.63 [.32, 1.24]	.63 [.32, 1.24]	.61 [.30, 1.22]
Father's alive	2.02 [.98, 4.16]	1.67 [.66, 4.25]	1.29 [.49, 3.37]	1.71 [.66, 4.40]	1.61 [.62, 4.16]	1.51 [.57, 3.95]
Mother’s alive	1.11 [.50, 2.47]	1.39 [.51, 3.76]	1.80 [.65, 4.94]	1.40 [.51, 3.83]	1.52 [.55, 4.19]	1.78 [.61, 5.14]
Type of residence (city)	1.08 [.83, 1.40]	1.35 [.92, 1.99]	1.49 [1.00, 2.22]*	1.44 [.97, 2.13]	1.43 [.96, 2.12]	1.48 [.99, 2.21]*
Type of residence (refugee camp)	.72 [.50, 1.05]	.77 [.47, 1.28]	.80 [.47, 1.36]	.93 [.55, 1.57]	.86 [.51, 1.44]	.94 [.55, 1.59]
Type of residence (Village)	1.11 [.82, 1.52]					
Citizenship	1.01 [.78, 1.32]	1.16 [.82, 1.64]	1.16 [.82, 1.66]	1.22 [.86, 1.74]	1.18 [.83, 1.68]	1.21 [.85, 1.73]
**Exposure to war-traumatic events**						
Personal trauma	1.36 [1.26, 1.47]***		1.37 [1.25, 1.50]***			
Witnessing trauma of others	1.14 [1.08, 1.20]***			1.14 [1.08, 1.21]***		
Seeing properties demolition	1.30 [1.19, 1.42]***				1.30 [1.18, 1.44]***	
Overall trauma	1.10 [1.07, 1.14]***					1.11 [1.07, 1.14]***
***R*^2^**		0.046	0.123	0.081	0.090	0.111

***p < .001, **p < .01, *p < .05.

## Discussion

The aim of this study was to investigate the prevalence of war-traumatic events and PTSD among children and adolescents following the November 2012 Israeli attacks on Gaza. To our knowledge, this is the first study utilizing the diagnostic criteria of PTSD according to DSM-V. It is the second major war that children and adolescents have experienced (the first happened in December 2008 to January 2009), in addition to intermittent military attacks by Israeli army forces that take place from time to time. Hence, Palestinian children and adolescents are living in a situation of ongoing trauma that makes their lives more difficult and leaves them potentially vulnerable to develop mental health problems. Therefore, we intended to assess the effect of the exposure to the most recent (2012) war-related traumatic events on children and adolescents' mental health, specifically PTSD and daily functioning. The results showed that every child or adolescent had been exposed to at least one war-traumatic event. Further, the prevalence of exposure to traumatic war event categories (experiencing personal trauma, witnessing trauma to others, and seeing demolition of properties) was high; at least 84% of the participants experienced all types of exposure. As a result, a large proportion (54%) of participants developed PTSD symptoms according to DSM-V. These findings are consistent with previous studies showing that the greater the exposure to traumatic events, the greater the likelihood of developing PTSD symptoms (10, 21–23, 26, 28, 31, 41).

Undoubtedly, increased frequency and severity of war can cause severe symptoms and, in particular, PTSD symptoms. Accordingly, it is not unexpected that Palestinian children and adolescents who experienced continuous exposure to war-traumatic events reported increased prevalence of intrusion symptoms, avoidance, alterations in arousal and reactivity, and negative alterations in cognitions and mood. Consequently, the prevalence of PTSD found to be elevated in this study compared to previous studies (26, 42, 43). In addition, the results were consistent with previous studies showing high association between exposure to war-traumatic events and PTSD criteria (42–46). Exposure to war-traumatic events was associated not only with elevated level of PTSD criteria, but also with impairments in many areas of functioning. These findings are in-line with previous studies indicating that exposure to war-traumatic events is associated with impairments in cognitive, emotional, social, and academic functioning (42, 47, 48) and somatic symptoms (3, 42, 49).

Similar to previous studies (50–53), boys reported more exposure to war-traumatic events than girls. The reason of this may be related to cultural issues as Palestinian boys are typically encouraged to participate in political activities during the war, while girls are not (54).

Another factor that can affect exposure to war-traumatic events is the age of children. Results indicated that older children report higher levels of exposure to war-traumatic events, confirming what other studies of exposure to traumatic events have also shown (5, 7, 46). Palestinian children feel that it is their responsibility to participate in community activities. As they get older this responsibility increases. Accordingly, the more that children and adolescents participate in community activities, the more exposure to war-traumatic events they experience.

Children and adolescents living in villages showed more exposure to war-traumatic events than those living in cities or refugee camps. One of the explanations is that the villages are closest in proximity to areas of attack at the time of war. Haj-Yahia (55) found that children who are living in villages showed more internalizing and externalizing symptoms as a result of exposure to traumatic events than those living in cities or refugee camps.

In regard to PTSD diagnosis, although many studies have shown females exhibit more PTSD symptoms than males (28, 42, 46, 48), the current finding is consistent with other studies that found no gender differences in relation to PTSD prevalence (56, 57). However, being a female was a risk factor of PTSD symptoms and PTSD diagnosis according to DSM-V.

The results also demonstrated that PTSD was significantly associated with age. Older children exhibited more PTSD symptoms than younger ones. The cause may be related to biological and emotional changes that occur in this period of life; these changes are considered as stressors and may contribute to the instability of adolescent mental and physical health at time of stressful events such as war trauma. Thus, Gaza adolescents face not only internal stressors caused by biological and emotional changes but also external stressors due to exposure to war-related traumatic events, which make them more likely to develop PTSD symptoms.

Confirming findings from many studies that have revealed that low socioeconomic status is one of the factors that can increase the likelihood of PTSD development (26, 28, 58), the current results showed that children and adolescents with unemployed mothers and with parents with low educational levels were more likely to develop PTSD compared to those with employed mothers and parents with higher educational levels. It can be speculated that unemployed mothers and parents with lower educational levels may have lower family income and more economic pressure. Consequently, these may cause a reduction of resources that could otherwise serve to buffer the impact of war traumatic events on children's mental health. In addition, parents can play a central role in equipping their children with suitable resources fostering the development of resilience and coping strategies that can help them bounce back and prevent the development of PTSD.

This study investigated how different types of war trauma affected the development of PTSD while taking into account other demographic and socioeconomic status factors. Future studies may focus on investigating trauma more in depth by using a qualitative method. Challenges in the Gaza Strip should also be taken into account in further studies, since PTSD could arise due to other variables occurring between the war in 2012 and the time of the study.

## Limitations of the Study

This study has several limitations. Firstly, data were collected a year after the war-related traumatic events occurred. As a result, participants may have forgotten some information regarding the emotional and social effects of the war. However, research has found that exposure to war-traumatic events predicted PTSD six decades after the war (59). Secondly, data were collected via multiple-choice questionnaires, not narrative answers. Future studies should use clinical assessment for more accurate diagnosis of PTSD. Thirdly, data were collected from one source (school pupils). Future studies should consider collecting data from other sources such as parents and teachers.

## Conclusion

To our knowledge, this is the first study that investigated the relationship between demographic, socioeconomic status and different types of war trauma in one hand, and PTSD diagnosis according to DSM-V on the other hand, after the war in 2012 in the Gaza Strip.

Despite of the previous limitations, this study provides valuable evidence that demographic and socioeconomic factors mediate the relationship between war traumatic events and its classifications and categories, and PTSD symptoms and diagnosis. The current study proves that the surrounding environment of the child has an influence on the development of PTSD either as a risk or as a protective factor. The application of the ecological framework theory with children exposed to difficult situations as here involve the relationships between risk and protective factors in the various levels of the ecological model which are the individual (e.g., age, gender), family (e.g., family size, SES), and environment (type and place of residence, citizenship, war trauma and political situation) (60). As a result, the core stone of the ecological model represented by the connectedness of these resources and factors and their implications on the individual's life. Thus, the children who are exposed to war traumatic events as in this study, the risk to develop PTSD may exaggerate if the extended resources such as their familial environment are affected by war-related trauma (61, 62). For example, the findings emphasize the importance of parental level of education and income, which can, in turn, mitigate the effects of exposure to traumatic event and thus reduce the probability to be diagnosed with PTSD. Thus, intervention programs should focus and take into account the background of the diagnosed child including their gender, age, where they live, and their socioeconomic status (e.g., family income, parents' educational level, family size). Ideally, intervention programs should be designed to alleviate the psychological symptoms and to enhance the resilience of the children. Strategies, such as psychodrama, painting, and role playing, could be used with the children as well as social support for parents and teachers.

## Data Availability Statement

The datasets generated for this study are available on request to the corresponding author.

## Ethics Statement

The studies involving human participants were reviewed and approved by the Ethical committee of Kingston University London. Written informed consent to participate in this study was provided by the participants' legal guardian/next of kin.

## Author Contributions

BE-K conceived of the study and its design, and coordinated and drafted the manuscript. MS conceived of the study and its design, and coordinated and drafted the manuscript. CA has been involved in drafting the manuscript. All authors read and approved the final manuscript and revised it critically for important intellectual content.

## Funding

This work was supported by the Qatar National Research Fund (QNRF), a member of Qatar Foundation Doha, Qatar, NationalPriority Research Programs (NPRP) under Grant (NPRP 7 - 154 - 3 - 034) funded to Professor MS.

## Conflict of Interest

The authors declare that the research was conducted in the absence of any commercial or financial relationships that could be construed as a potential conflict of interest.
